# Unphysical Critical Curves of Binary Mixtures Predicted with GERG Models

**DOI:** 10.1007/s10765-020-02743-3

**Published:** 2016

**Authors:** Ulrich K. Deiters, Ian H. Bell

**Affiliations:** 1 Institute of Physical Chemistry, University of Cologne, Greinstr. 4–6, 50939 Cologne, Germany; 2 Applied Chemicals and Materials Div., National Institute of Science and Technology, Boulder, CO 80305, USA

**Keywords:** Asymmetric fluid mixtures, Critical curves, GERG equation of state, Vapor–liquid equilibria

## Abstract

When applied to asymmetric binary mixtures (e.g., methane + pentane or heavier alkanes, hydrogen-containing mixtures), the GERG equation of state (GERG-2004 or GERG-2008) predicts critical curves with physically unreasonable temperature maxima above the critical temperature of the heavier component. These maxima are associated with physically impossible vapor–liquid equilibria. The phenomenon is probably caused by corrections for critical anomalies that were built into the empirical pure-fluid equations of state forming the foundation of the GERG model. These corrections ensure that the model represents thermodynamic data of pure fluids quite well even close to their critical points. For mixtures, however, the corrections can cause artifacts.

## 1 Introduction

In 1989 Wagner and Setzmann published an equation of state for methane [1] that had an exceptionally wide range of validity and was able to represent almost all existing experimental data on methane within the error of the experiments. It became not only the reference equation for methane, but also the template for the reference equations of several other substances. Later Wagner, Span, and Lemmon generated simplified equations of state [2–5], to be used when computing speed was important or the experimental data were too scarce to permit the construction of a full reference equation.

The GERG-2004 equation of state and its upgrade GERG-2008 extended these pure-fluid equations to mixtures [6, 7]. We shall henceforth use the name “GERG” when referring to GERG-2008 or to features common to both versions. At the core of the GERG concept there is a “multifluid approach” that makes it possible to make use of pure-fluid equations of state even if they have different numbers of parameters, or even different mathematical structures. The GERG equation allows consistent and remarkably accurate calculations of natural gas properties, from single-phase properties like volumetric data or speed of sound to vapor–liquid phase equilibria.

A difficulty with the GERG equation is that its underlying pure-fluid equations of state have—or may have—density and temperature ranges where they return unphysical values. This makes it difficult to apply algorithms for the calculation of phase equilibria or particularly critical curves which need to scan ranges of thermodynamic states. Fortunately it was possible to develop algorithms for critical curves that avoid this difficulty, for instance the method of Bell and Jäger [8] or, more recently, the method of Deiters and Bell [9]. The latter uses differential equations to express the critical conditions, and then obtains critical curves by integrating the resulting initial-value problem.

It is therefore possible to calculate critical curves of mixture with the GERG equation. Systematic studies, however, revealed a peculiar problem that will be described in the next section.

## 2 The Problem

**Fig. 1:**
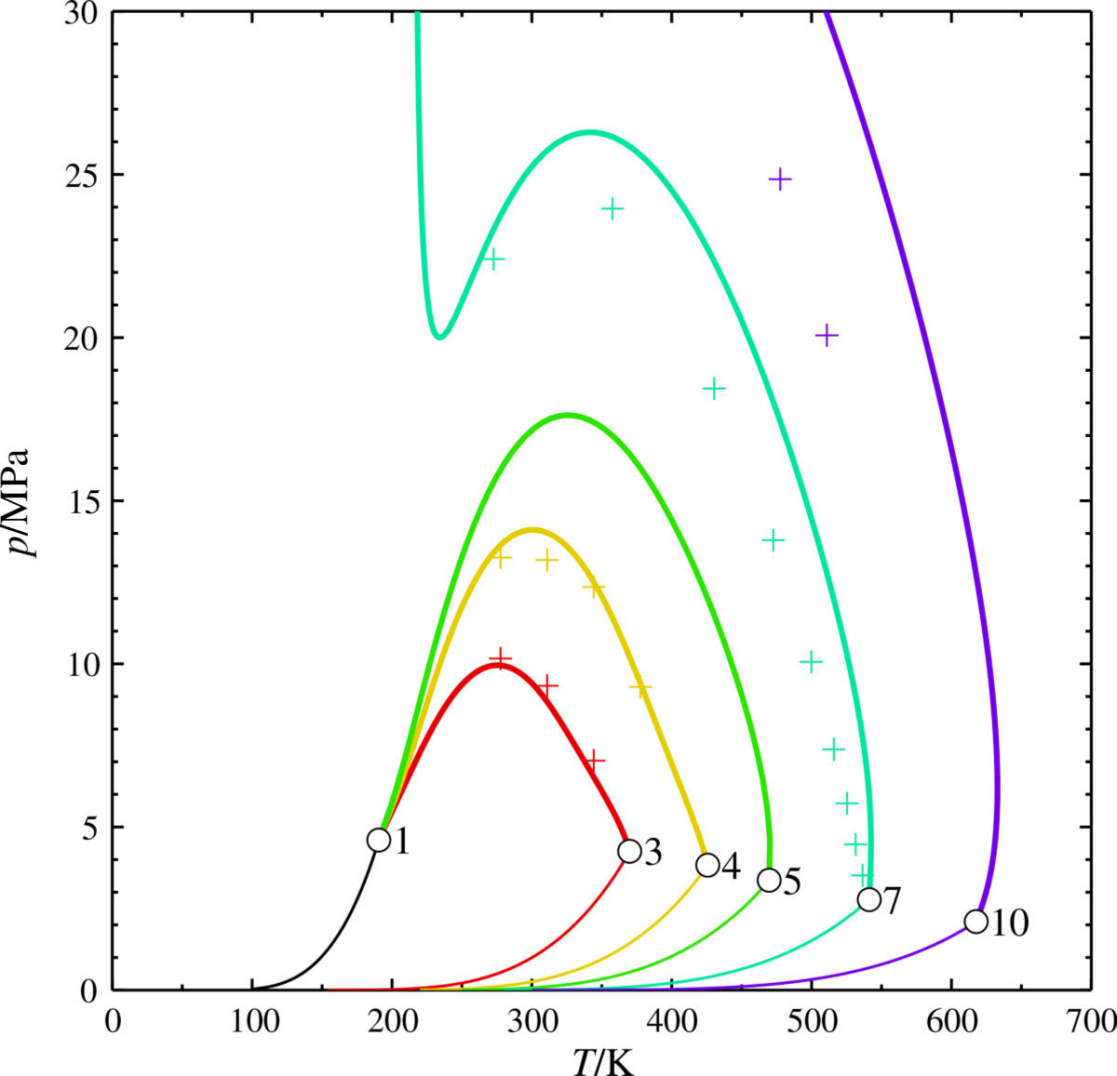
Critical curves of the mixtures (CH_4_ + C_*n*_H_2*n*+2_) with *n* = 3, 4, 5, 7, 10, calculated with the GERG model. 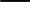 critical curve, 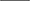 vapor pressure curve, ○ pure-component critical point, + experimental data [12–15], parameter: carbon number *n*

Figure 1 shows *pT* projections of the critical curves of mixtures of methane with ethane, propane, pentane, heptane, or decane, all calculated with the GERG model. The systems (methane + propane), (methane + butane) and (methane + pentane) exhibit an uninterrupted vapor–liquid critical curve connecting the pure-component critical points; they belong to phase diagram Class I according to the classification of van Konynenburg and Scott [10], or Class 1^P^ according to the rational nomenclature of Bolz et al. [11]. The other two systems belong to Class III or 1^C^1^Z^, respectively.

**Fig. 2:**
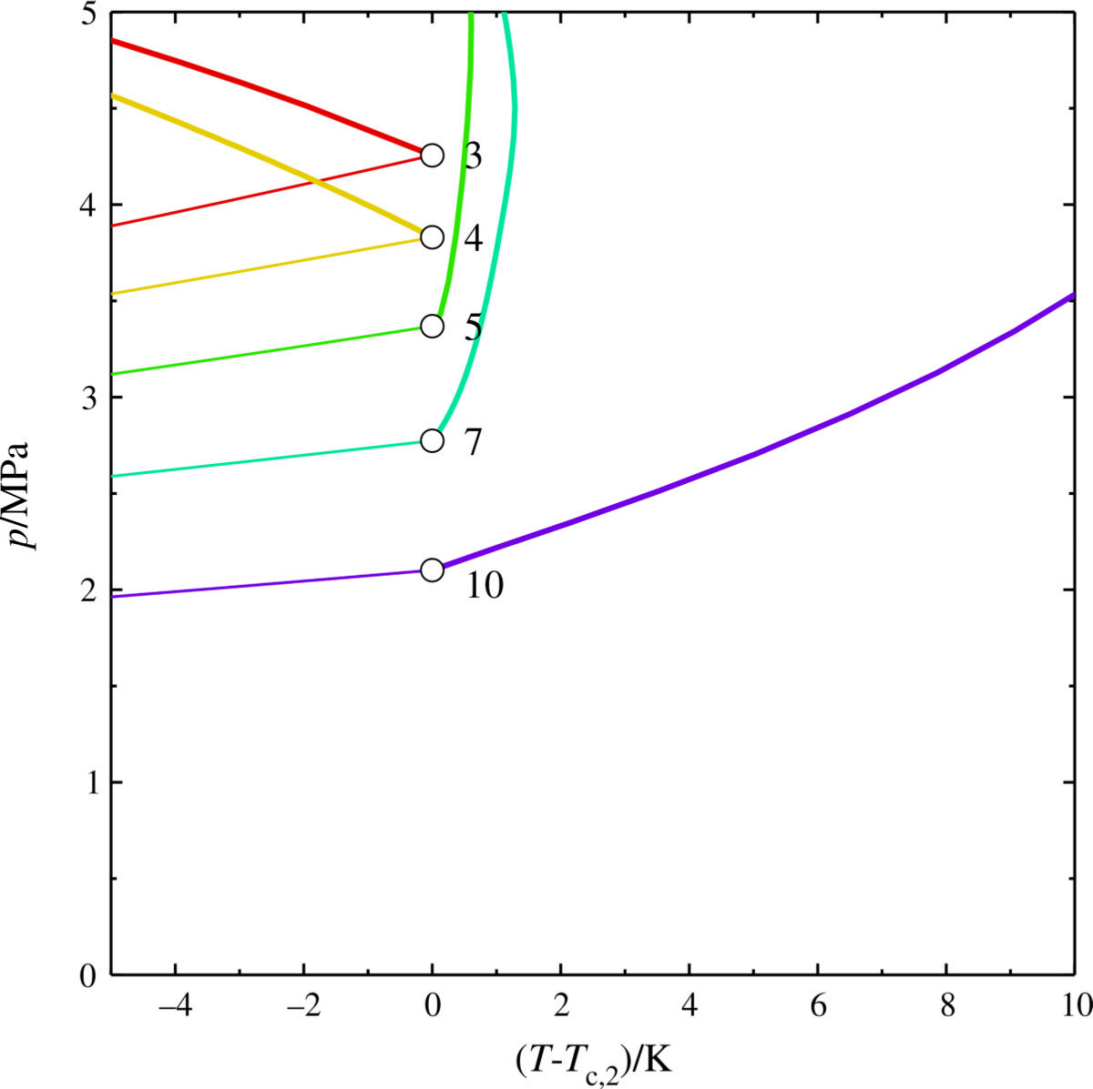
Critical curves of the mixtures (CH_4_ + C_*n*_H_2*n*+2_) with *n* = 3, 4, 5, 7, 10, calculated with the GERG model; enlargement of the region of the critical point of the second component. 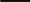 critical curve, 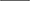 vapor pressure curve, ○ pure-component critical point, parameter: carbon number *n*

A close inspection of the region close to the critical point of the heavier component (Fig. 2) reveals an unusual feature: The predicted critical curves of (methane + pentane) to (methane + decane) originate at the critical point of the second component with a positive slope and pass through a temperature maximum. This is also the case for (methane + butane), but here the maximum is too close to the critical point to be visible at the resolution of the diagram. The behavior is not in agreement with experimental data [12–15], which clearly exhibit a negative slope.

This is a rather surprising result. Vapor–liquid equilibria above the critical temperature of the less volatile component are possible, in principle, but merely in two cases, namely

- Negative azeotropy:
  This usually requires large negative deviations from Raoult’s Law. Such deviations are caused by strong attractive cross interactions between the mixture components, like, e.g., in the system (H_2_O + HBr). Jaubert and Privat [16] showed that positive deviations from Raoult’s Law can cause negative azeotropy, too, but then the excess Gibbs energy (as a function of composition) must have regions where its slope varies very strongly, and this usually requires dominant chemical interactions.
  But neither large negative nor strongly varying positive excess Gibbs energies can be expected for mixtures where the components have similar chemical constitutions.
- Gas–gas equilibria of the 1st kind:
  Such equilibria exist in mixtures in which the components have rather weak cross interactions. A famous example is the system (He + Xe) [17].
  Again, such weak cross interactions are untypical for alkane mixtures. Furthermore, in gas–gas equilibria of the 1st kind, the critical curve originating a the critical point of the less volatile component has a positive slope (in a *pT* diagram) and does not pass through a temperature maximum.

The calculation of critical curves of fluid mixtures from an equation of state is known to be a rather demanding task, and one might therefore wonder if the unusual shape of the critical curve is a numerical artifact or perhaps even caused by a programming error. In such a case it is advisable to inspect the object functions of the underlying mathematical problem. These are the critical conditions, expressed in the formalism of isochoric thermodynamics [9, 18],

(1) $$\lambda_{\min}(\rho ,T)\;=0\;\frac{\text{d}\lambda_{\min}\left(\rho +\alpha u_{\min},T\right)}{\text{d}\alpha }=0.$$

Here *λ*_min_ denotes the lowermost eigenvalue of **Ψ**, the Hessian matrix of the Helmholtz energy density Ψ(***ρ***, *T*) ≡ *ρA*_m_(***ρ***, *T*),

(2) $$\Psi =\left(\begin{array}{ccc} \Psi_{11} & \ldots & \Psi_{1N}\\ \vdots & \ddots & \vdots \\ \Psi_{N1} & \ldots & \Psi_{NN} \end{array}\right)\;\text{with}\;\Psi_{ij}={\left(\frac{\partial^{2}\Psi (\rho ,T)}{\partial \rho_{i}\partial \rho_{j}}\right)}_{T},$$

and ***u***_min_ the associated eigenvector. The second criterion may be regarded as a directional derivative of *λ*_min_ in the direction of this eigenvector. ***ρ*** is the vector of molar concentrations, with *ρ*_*i*_ =*ρx*_*i*_ (total molar density times mole fraction of component *i*).

The critical conditions Eq. (1) were originally proposed by Quiñones-Cisneros, although for a slightly differently defined Hessian matrix [19, 20]. This approach offers advantages over the commonly used formulation of the critical conditions with determinants, which creates additional, unstable solutions.

**Fig. 3:**
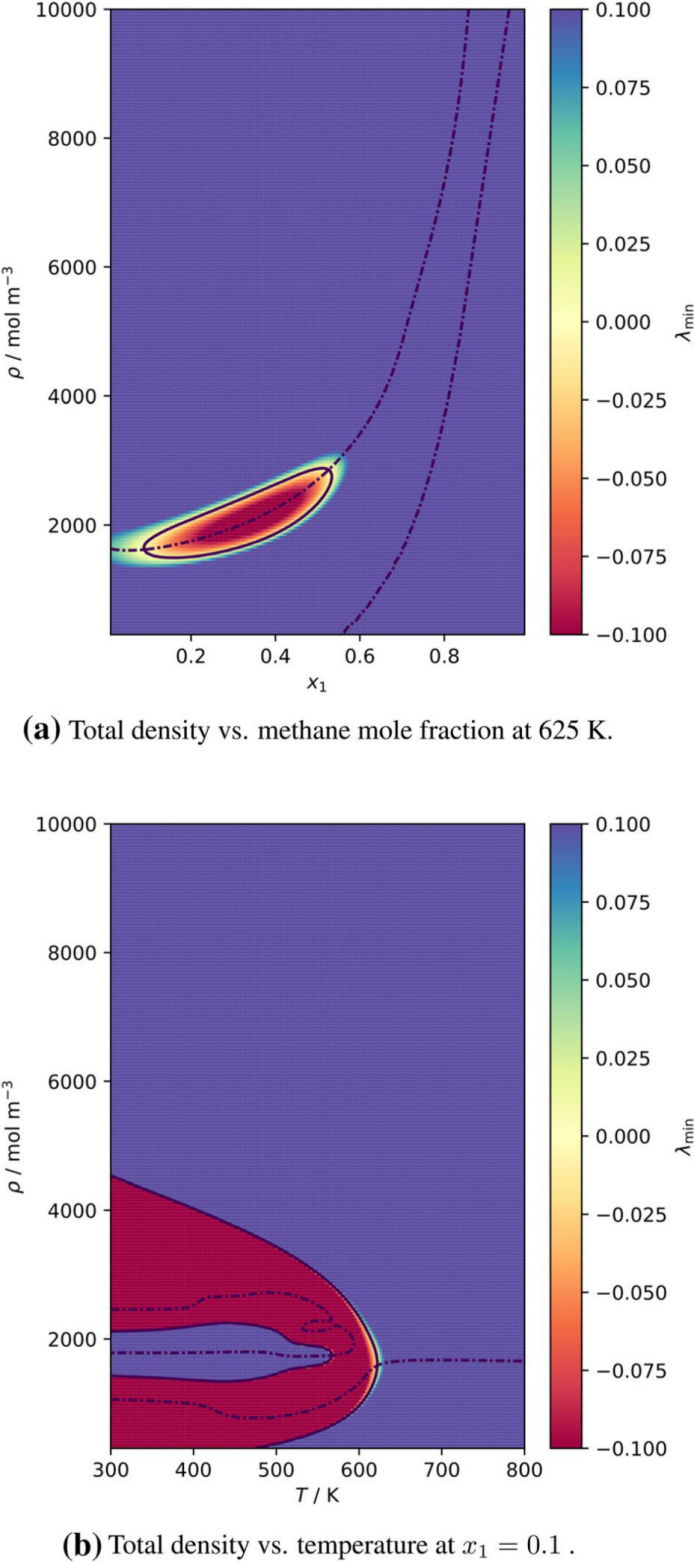
Critical conditions of the (methane + decane) system, calculated with the GERG model. Background color: value of *λ*_min_, — locus of *λ*_min_ = 0, –.– locus of d*λ*_min_∕d*α* = 0 (cf. Eq. (1))

Figure 3 shows the behavior of *λ*_min_ of the (methane + decane) system close to the decane critical point. The *ρx*_1_ diagram (a) shows that the locus of the first critical condition forms a closed loop (already a rather peculiar phenomenon!) and intersects the locus of the second criterion twice—and that above the critical temperature of decane. The *ρT* diagram (b) reveals that the locus of the second criterion exhibits a rather surprising indentation. The unexpected shape of the critical curve is therefore not an artifact of the program that solves the critical conditions but a property of the Ψ(***ρ***, *T*) function of the GERG model.

**Fig. 4:**
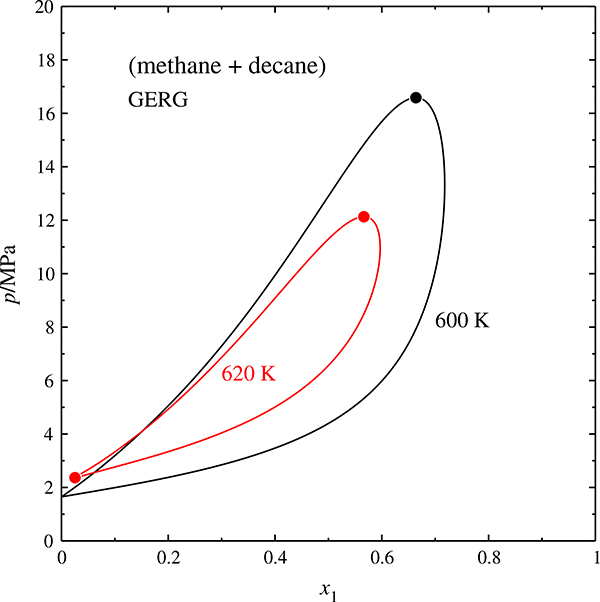
Isothermal phase envelopes of the (methane + decane) system, calculated with the GERG model, at 600 and 620 K. 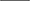 phase envelope, • binary critical point

A second, independent way of ascertaining that the predicted critical curve is not an artifact of the critical-curve calculator is the prediction of phase envelopes, which is done by a different program and a different algorithm. Figure 4 shows isothermal phase envelopes for the (methane + decane) system at 600 K (slightly below the critical point of decane) as well as 620 K (between the critical temperature of decane and the temperature maximum of the critical curve). The 600 K isotherm exhibits the typical loop shape of systems having one supercritical component. The 620 K isotherm has a similar shape, but does not touch the left ordinate. Liquid and vapor branch of the phase envelope come together at *x*_1_ ≈ 0.024, and this intersection point has mathematically the properties of a critical point.

It is evident that neither negative azeotropy nor a gas–gas equilibrium can be the cause of such a phase envelope, and we must conclude that the shape, and ultimately the existence, of the 620 K isotherm is physically not reasonable.

## 3 Searching for an Explanation

First of all, we must point out that the unphysical temperature maximum along the critical curve is not affecting the (methane + decane) system only: it appears for methane mixtures with butane, pentane, or heptane, too. The phenomenon appears to get more pronounced as the chain length of the heavier alkane gets longer.

### 3.1 Are the Unphysical Temperature Maxima Typical for Mixtures in Which Methane is the Light Component?

**Fig. 5:**
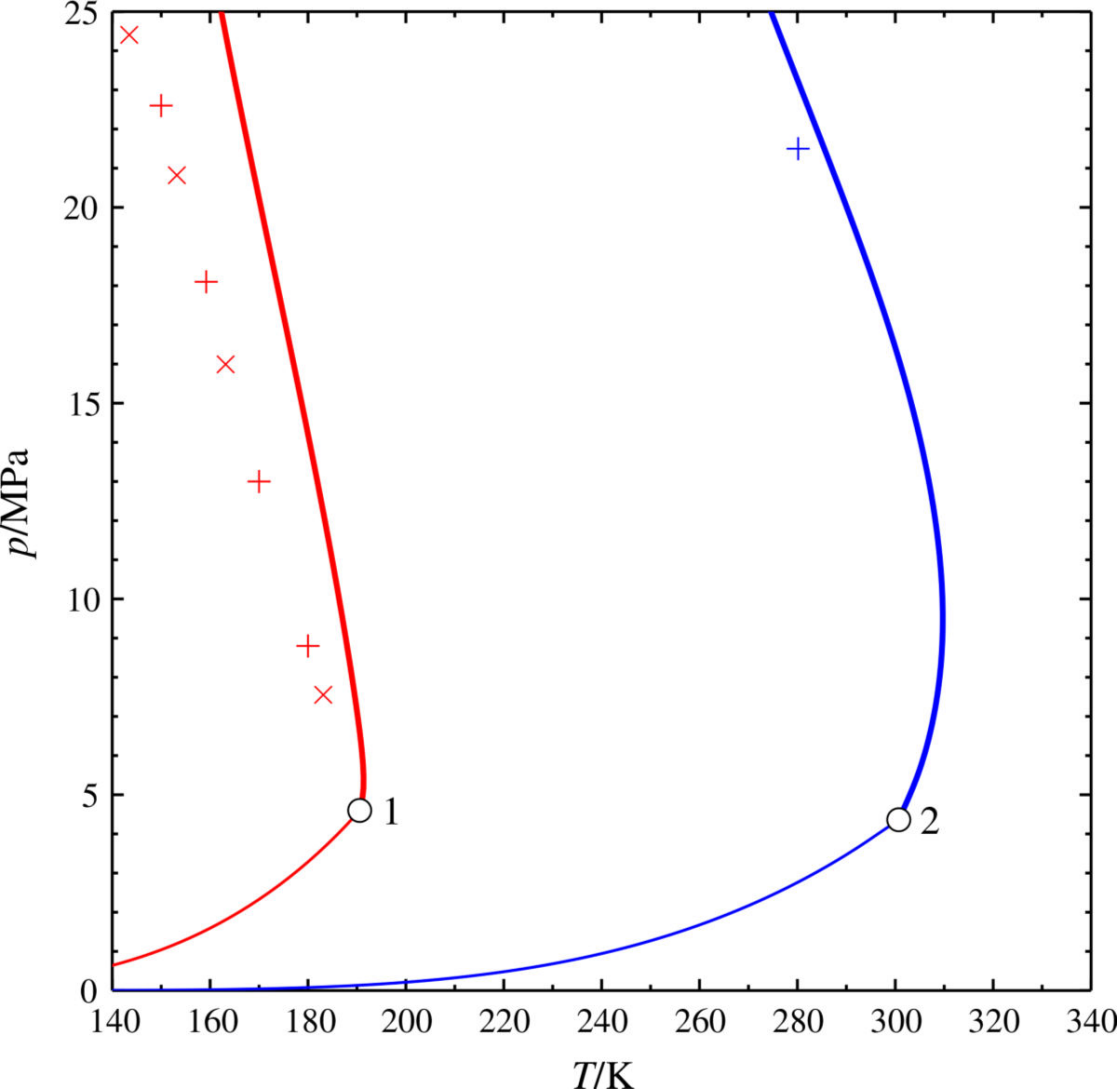
Critical curves of the mixtures (H_2_ + CH_4_) and (H_2_ + C_2_H_6_), calculated with the GERG model 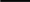 critical curve, 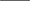 vapor pressure curve, ○ pure-component critical point, +, × experimental data [21–23], parameter: carbon number *n*

Figure 5 shows that this not not the case. Mixtures with hydrogen as the light component show the same unphysical behavior. Again, the experimental data for the mixtures shown here, (hydrogen + methane) [21, 23] and (hydrogen + ethane) [22] do not show any unusual behavior.

A temperature maximum along the critical curve was also observed for the (ethane + decane) system (diagram not shown here).

### 3.2 A re the Unphysical Temperature Maxima Caused by an Inferior Equation of State for Methane?

The GERG model makes use of pure-fluid equations of state for the mixture components. The multifluid approach, which the GERG model uses, permits combining equations of state with different numbers of terms, or even different structures.

The original GERG models contain a simplified equation of state for methane [2]. One might therefore wonder if a better methane equation would make the unphysical maximum vanish. We therefore substituted the methane reference equation of Wagner and Setzmann [24] for the simplified equation and repeated the calculations of the critical curves for (methane + decane) and for (hydrogen + methane).

It turned out that this substitution did not change the critical curve of the (methane + decane) system perceptibly. For the (hydrogen + methane) system the changes are negligible, too, except in the immediate vicinity of the critical point of methane. The unphysical temperature maximum was not affected by the substitution.

We must therefore conclude that using the simplified equations of state instead of reference equations is not responsible for the unphysical temperature maximum.

### 3.3 I s the Departure Function Responsible?

The GERG model obtains the dimensionless residual Helmholtz energy $\left(\alpha^{\text{r}}=A_{\text{m}}^{\text{r}}/(RT)\right)$ of a mixture from

(3) $$\alpha^{\text{r}}(\omega ,\tau ,x)=\sum_{i=1}^{N}x_{i}\alpha_{0i}^{\text{r}}(\omega ,\tau )+\sum_{i=1}^{N}\sum_{j=i+1}^{N}x_{i}x_{j}F_{ij}\alpha_{ij}^{\text{r}}(\omega ,\tau ),$$

where $\alpha_{0i}^{\text{r}}(\omega ,\tau )$ is the dimensionless residual Helmholtz energy of the pure component *i* and $\alpha_{ij}^{\text{r}}(\omega ,\tau )$ the so-called departure function of the *ij* pair of components. The latter can be fitted to experimental data, if enough are available; otherwise it can be turned off by setting the switch parameter *F*_*ij*_ to zero. *ω* and *τ* are the mean reduced density and the mean reduced inverse temperature, respectively.

In many of our calculations the departure function had been turned off, but it did not solve the problem. We must therefore conclude that the unphysical temperature maxima are not caused by the departure function.

### 3.4 A re the Asymmetric Mixing Rules in the GERG Model Responsible?

The reduced density and the reduced temperature appearing in Eq. (3) are defined as

(4) $$\omega =\frac{\rho }{\rho_{\text{r}}}\;\text{and}\;\tau =\frac{T_{\text{r}}}{T},$$

where *ρ* and *T* are the molar density and the temperature of the mixture. The reducing properties *ρ*_r_ and *T*_r_ are functions of composition and the pure-fluid critical properties,

(5) $$\frac{1}{\rho_{\text{r}}}=\sum_{i=1}^{N}\frac{x_{i}^{2}}{\rho_{\text{c},i}}+\sum_{i=0}^{N}\sum_{j=i+1}^{N}\frac{2x_{i}x_{j}\beta_{V,ij}\gamma_{V,ij}\left(x_{i}+x_{j}\right)}{\beta_{V,ij}^{2}x_{i}+x_{j}}\cdot \frac{1}{8}{\left(\rho_{\text{c},i}^{-1/3}+\rho_{\text{c},j}^{-1/3}\right)}^{3},\;\;T_{\text{r}}=\sum_{i=1}^{N}x_{i}^{2}T_{\text{c},i}+\sum_{i=0}^{N}\sum_{j=i+1}^{N}\frac{2x_{i}x_{j}\beta_{T,ij}\gamma_{T,ij}\left(x_{i}+x_{j}\right)}{\beta_{T,ij}^{2}x_{i}+x_{j}}\cdot \sqrt{T_{\text{c},i}T_{\text{c},j}}.$$

As a first test we replaced this pair of mixing rules by van der Waals mixing rules,

(6) $$\rho_{\text{r}}=\sum_{i=1}^{N}x_{i}\rho_{\text{c},i}\;T_{\text{r}}\rho_{\text{r}}=\sum_{i=1}^{N}\sum_{j=1}^{N}\frac{1}{2}x_{i}x_{j}\left(1-k_{ij}\right)\sqrt{T_{\text{c},i}T_{\text{c},j}}\left(\rho_{\text{c},i}+\rho_{\text{c},j}\right)$$

The adjustable parameter *k*_*ij*_ was set to 0.18 for the (methane + decane) system.

As a second test we used the Peng–Robinson equation of state [25, 26] and replaced its usual mixing rules with the GERG asymmetric rules, keeping the mixing rule parameters. As the Peng–Robinson *a* and *b* parameters (energy parameter and covolume) are proportional to *T*_c_*ρ*_c_ and *ρ*_c_, respectively, the conversion between *a*, *b* and *T*_c_, *ρ*_c_ parameter sets is easily accomplished.

**Fig. 6:**
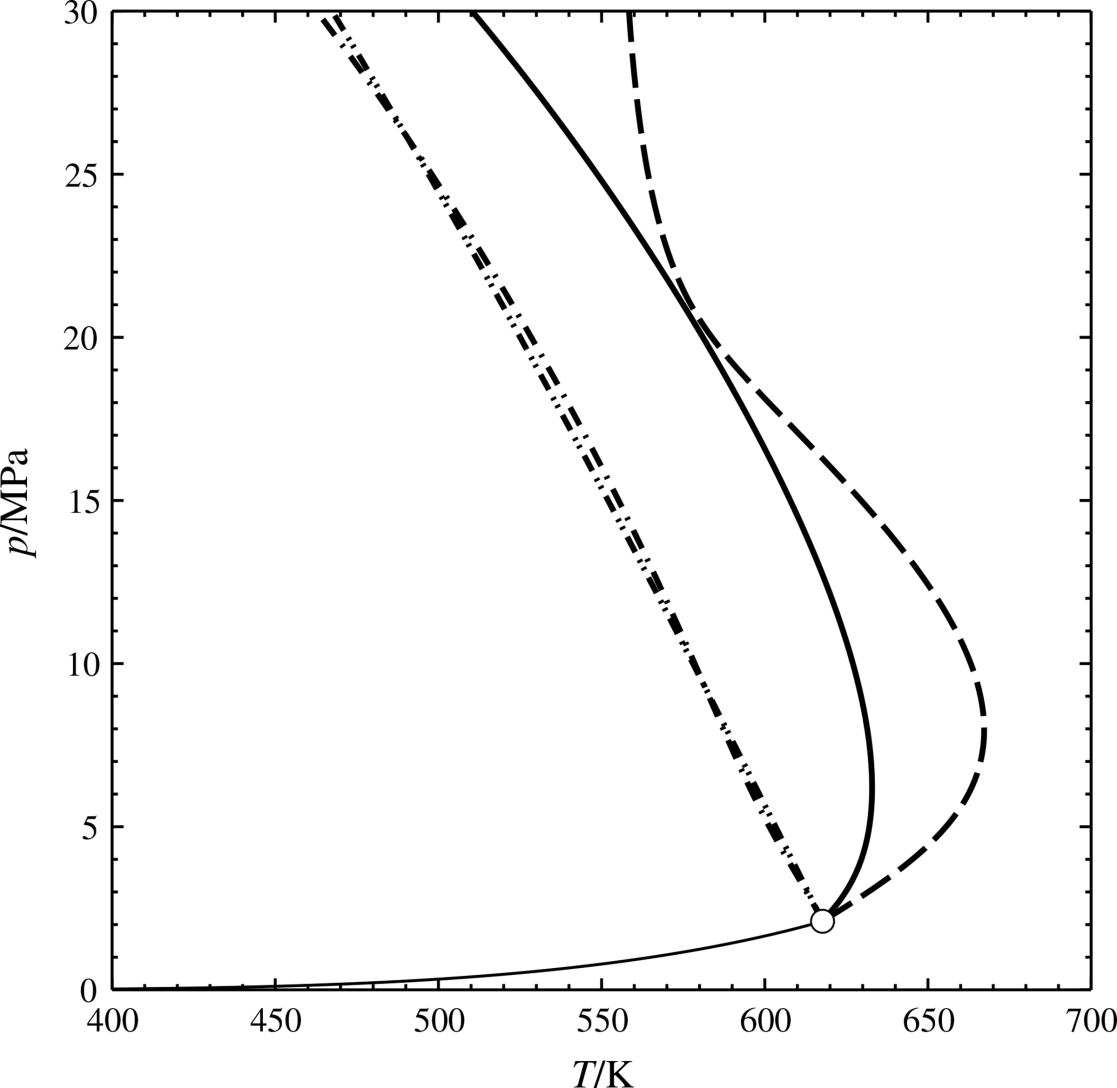
Critical curve of the (methane + decane) system (high-temperature portion). 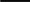 original GERG2008, – – –modified GERG with van der Waals mixing rules, − · − · −Peng–Robinson equation with asymmetric mixing rules, − · · − ··Peng–Robinson equation with van der Waals mixing rules, 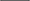 vapor pressure curve of decane, ○ decane criticl point

The results are presented in Fig. 6. It turns out that the unphysical temperature maxima appear irrespective of the mixing rule whenever the GERG pure-fluid equations of state are used. We must therefore conclude that the asymmetric mixing rules are not the cause of those maxima.

## 4 Critical Anomalies

At this point it appears that the unwanted behavior can be traced to the pure-fluid equations built into the GERG model. These are either reference equations of the Wagner–Setzmann type [1] or simplified versions developed by Wagner, Span, and Lemmon [2–5]. This is surprising, because these equations of state can represent all thermodynamic properties of pure fluids with surpassing accuracy.

But perhaps this accuracy is at the root of the problem. It is known that, close to a critical point, density fluctuations cause the so-called critical anomalies—departures of thermodynamic functions which are often described with power laws in which exponents with non-classical values appear. It is known in particular that the fundamental equation of a fluid must be non-analytical at the critical point, and that no analytical equation can accurately represent the behavior of fluids. The equations of state underlying the GERG model are analytic, but because of their clever structure and the many adjustable parameters they can account for the critical anomalies rather well, except for a tiny region around the critical point—the critical point of a pure fluid. The critical states of mixtures, however, obey different rules. They are not characterized by a divergence of the isothermal compressibility (except for critical azeotropy). Instead, the criticality of a mixture is related to the vanishing local curvature of the Helmholtz energy density surface as a function of the concentrations of the components, and the critical anomalies are caused by density fluctuations of all components.

It is therefore conceivable that the empirical corrections for critical anomalies, which were built into the equations of the state underlying the GERG model, are not merely superfluous, but even noxious for mixtures.

This is of course merely a conjecture. But unexpected side-effects of the empirical corrections for critical anomalies have been observed before, name in the shape of isentropic expansion curves [27, Figs. 17–18].

### 4.1 The Influence of Pure-Fluid Critical Corrections on the Critical Curves Of Mixtures

At this point it is necessary look at the way how the behavior of a pure fluids near to its critical point are usually described. In this region the thermodynamic functions can be approximated by power laws, for instance

(7) $$C_{V\;\text{m}}=RB_{C}|\delta \tilde{T}{|}^{-\alpha }$$

for the isochoric heat capacity along the critical isochore^1^,

(8) $$\left|\rho^{\phi }-\rho_{\text{c}}\right|=\pm B_{\rho }\rho_{\text{c}}{\left(-\delta \tilde{T}\right)}^{\beta }$$

for the orthobaric densities (*ϕ* = liquid | vapor) along the vapor–liquid phase envelope (*T* ≤ *T*_c_),

(9) $$\left|\kappa_{T}^{-1}\right|=B_{K}RT_{\text{c}}\rho_{\text{c}}|\delta \tilde{T}{|}^{\gamma }$$

for the isothermal compressibility along the critical isochore, and

(10) $$\left|p-p_{\text{c}}\right|=B_{p}RT_{\text{c}}\rho_{\text{c}}{\left|\frac{\rho -\rho_{\text{c}}}{\rho_{\text{c}}}\right|}^{\delta }$$

along the critical isotherm. Here $\delta \tilde{T}\equiv \left(T-T_{\text{c}}\right)/T_{\text{c}}$ denotes a dimensionless temperature deviation, and *B*_*C*_, *B*_*ρ*_, *B*_*κ*_, and *B*_*p*_ are the so-called critical amplitudes. Eqs. (7) and (9) can also be used for subcritical temperatures if proper averages of the heat capacities or compressibilities, respectively, of the coexisting phases are used. In principle one should distinguish between critical amplitudes for *T* < *T*_c_ and *T* > *T*_c_, but they are usually assumed to be the same, unless very unusual symmetry conditions exist [28].

**Fig. 7:**
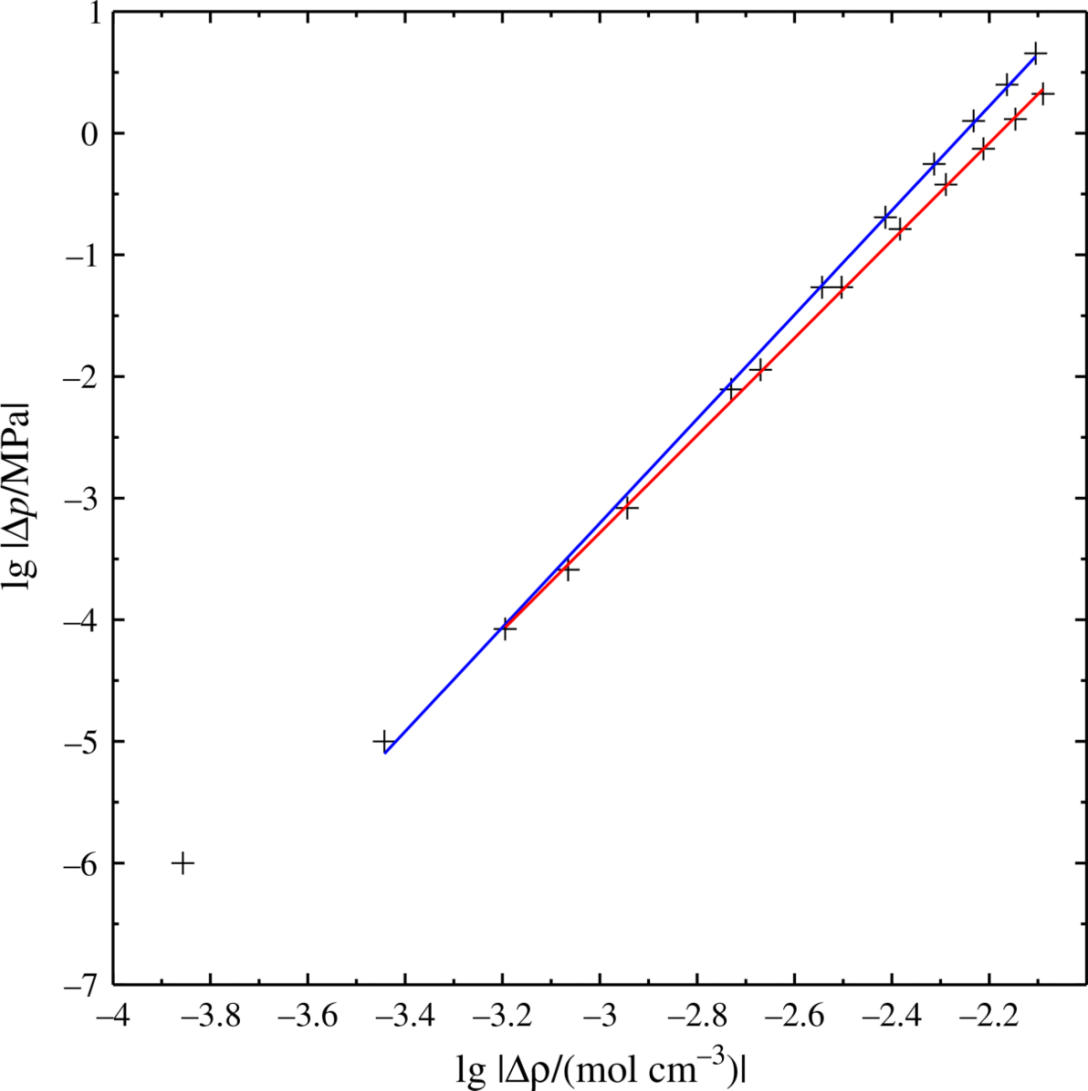
Pressure deviations |*p*−*p*_c_| vs. density deviations |*ρ*−*ρ*_c_| along the critical isotherm of methane, calculated from the methane reference equation of Setzmann and Wagner [24]. + reference equation, 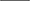 linear approximation (blue: liquid, red: vapor branch)

Cubic equations of state invariably yield the classical values for the critical exponents, namely *α* =0, $\beta =\frac{1}{2}$, *γ* = 1 and *δ* = 3. Experiments, however, yield *α* ≈ 0.1, *β* ≈ 0.34, *γ* ≈ 1.2, and *δ* ≈ 4.2. The pure-fluid equations of state within the GERG equation represent the experimental data quite well. Therefore they exhibit the same non-classical critical exponents—except for a very narrow region around the critical point, where they must revert to the classical behavior. An example is shown in Fig. 7, a double-logarithmic plot of the pressure and density deviations from the critical point along the critical isotherm: The slopes of the linear regions approximately amount to 4.2, the expected nonclassical value of the critical exponent *δ*.

It suggests itself to turn off terms of a Wagner–Setzmann or Wagner–Span equation one by one and to check whether this eliminates the unphysical behavior. Unfortunately, all the terms of such equations depend upon each other, so that such a course is not feasible.

As an alternative we construct a simple equation of state in which the near-critical corrections are separated from the rest. We give here the equations for the residual Helmholtz energy and the compression factor,

(11) $$\begin{array}{l} \frac{A_{\text{m}}^{\text{r}}}{RT}=-\ln(1-\xi )-\frac{8T^{*}}{T}\left[\frac{1}{c}\ln(1+c\xi )+\frac{1}{\xi^{\prime }}\exp\left(-{\left(w\ln\xi^{\prime }\right)}^{2}\right)\sum_{k=4}^{k_{\max}}p_{k}{\left(\Delta \xi^{\prime }\right)}^{k}\right]\\ \frac{p}{\rho RT}=\frac{1}{1-\xi }-\frac{8T^{*}}{T}[\frac{\xi }{1+c\xi }+\exp\left(-{\left(w\ln\xi^{\prime }\right)}^{2}\right)\sum_{k=4}^{k_{\max}}p_{k}(k{\left(\Delta \xi^{\prime }\right)}^{k-1}\\ \;-\frac{1+2w^{2}\ln\xi^{\prime }}{\xi^{\prime }}{\left(\Delta \xi^{\prime }\right)}^{k})]\\ \;\text{with}\;\xi =v^{*}\rho ,\;\xi^{\prime }=V_{\text{mc}}\rho ,\;\text{and}\;\Delta \xi^{\prime }=\xi^{\prime }-1. \end{array}$$

This is an extended van der Waals equation. The first term on the right-hand side is the repulsive term that is common to all cubic equations of state. The first part of the attractive term contains the parameter *c* (first introduced by Fuller [29]), which makes it possible to match the critical temperature, pressure, *and* density of a substance. The remainder of the attraction term is a polynomial centered on the critical density and multiplied with a log-normal distribution, which serves as a damping function.

For our tests we calculated critical curves of the (hydrogen + methane) system, which is known to belong to phase diagram class III (rational nomenclature 1^C^1^Z^) [21, 30]. The parameters were obtained as follows:

- The parameters of pure methane were fitted to the critical isotherm (obtained from the methane reference equation [24]), using state points from the linear range in Fig. 7, and of course the critical point. Parameter sets were computed for various values of *k*_max_, namely 0 (i.e., without the Gaussian term), 4, 6, and 8.
- For hydrogen we fitted merely T* and v* to its critical temperature and pressure, and adopted the methane results for the remaining parameters, which is equivalent to a corresponding-states approach.
- For mixtures we used van der Waals mixing rules,

(12) $$\begin{array}{l} T^{*}v^{*}=\sum_{i}\sum_{j}x_{i}x_{j}T_{ij}^{*}v_{ij}^{*}\\ v^{*}=\sum_{i}x_{i}v_{i}^{*}, \end{array}$$

where *i* and *j* represent the mixture components and *x*_*i*_, *x*_*j*_ their mole fractions. The characteristic temperature of the cross interaction was obtained from the Berthelot rule,

(13) $$T_{ij}^{*}=\sqrt{T_{ii}^{*}T_{jj}^{*}}.$$

As no quantitative modeling of the (hydrogen + methane) system was intended, no attempt was made to optimize $T_{ij}^{*}$.

The parameters are listed in Table 1.

**Fig. 8:**
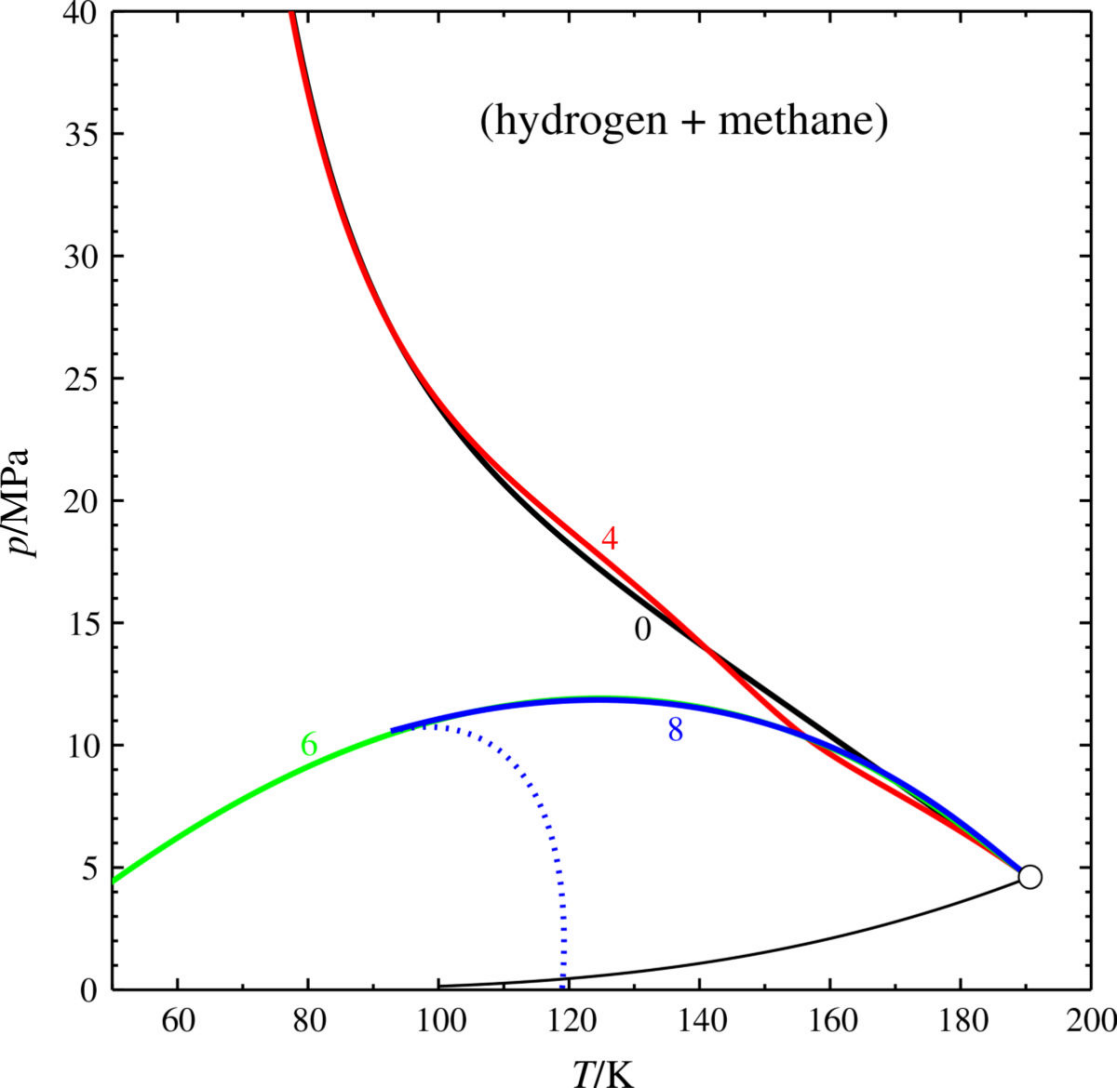
Critical curves of the (hydrogen + methane) system predicted with Eq. (11). 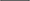 critical curve (parameter: max. order of the summation in the equation of state, *k*_max_), ○ critical point of methane

Figure 8 shows the critical curves of the (hydrogen + methane) system obtained with this equation of state. With *k*_max_ = 0, i.e., without the damped sum, the equation gives a very reasonable prediction of the critical curve; the phase diagram class is correct. For *k*_max_ = 4 the predicted phase diagram class is correct, too, but the critical curve exhibits a weird wriggle. For *k*_max_ = 6 a Class I phase diagram is wrongly predicted (rational classification: 1^P^). For *k*_max_ = 8 the critical curve turns unstable at about 90 K, which is physically unreasonable.

Incidentally, the equation of state could represent our *pVT* data set along the critical isotherm with an r.m.s. deviation of about 1.3 % for *k*_max_ = 0 or 4. With *k*_max_ = 6 a deviation of 0.1 % could be achieved, with *k*_max_ = 8 even 0.01 %. Therefore it turns out that the equation of state starts to predict wrong phase diagram classes as soon as it achieves a quantitative representation of the critical isotherm (which has an critical exponent of *δ* ≈ 4.2).

### 4.2 Widom Curves

Some thermodynamic properties, e.g., the isothermal compressibility, are infinite at the critical point of a pure fluid. Above the critical temperature, such properties exhibit maxima that become broader and dwindle with increasing temperature.

Other thermodynamic properties have inflection points at the critical point which can be observed at supercritical temperatures, too. The locus of such maxima or inflection points, respectively, on the *Tp* or *Tρ* plane is called a Widom curve. Evidently, there is more than one Widom curve, namely one for each thermodynamic property. All Widom curves, however, must originate at the critical point, and it turns out that—particularly in *pT* diagrams—they stay close to each other for some range of temperature.

The Widom curves can be used to distinguish regions of more liquid-like behavior from regions of gas-like behavior of supercritical fluids, and are therefore of some technical importance [31, 32]. Losey and Sadus used Widom curves for testing and comparing reference equations of state for Mie *n*–6 fluids [33].

Here we apply their method to the GERG equation.

**Fig. 9:**
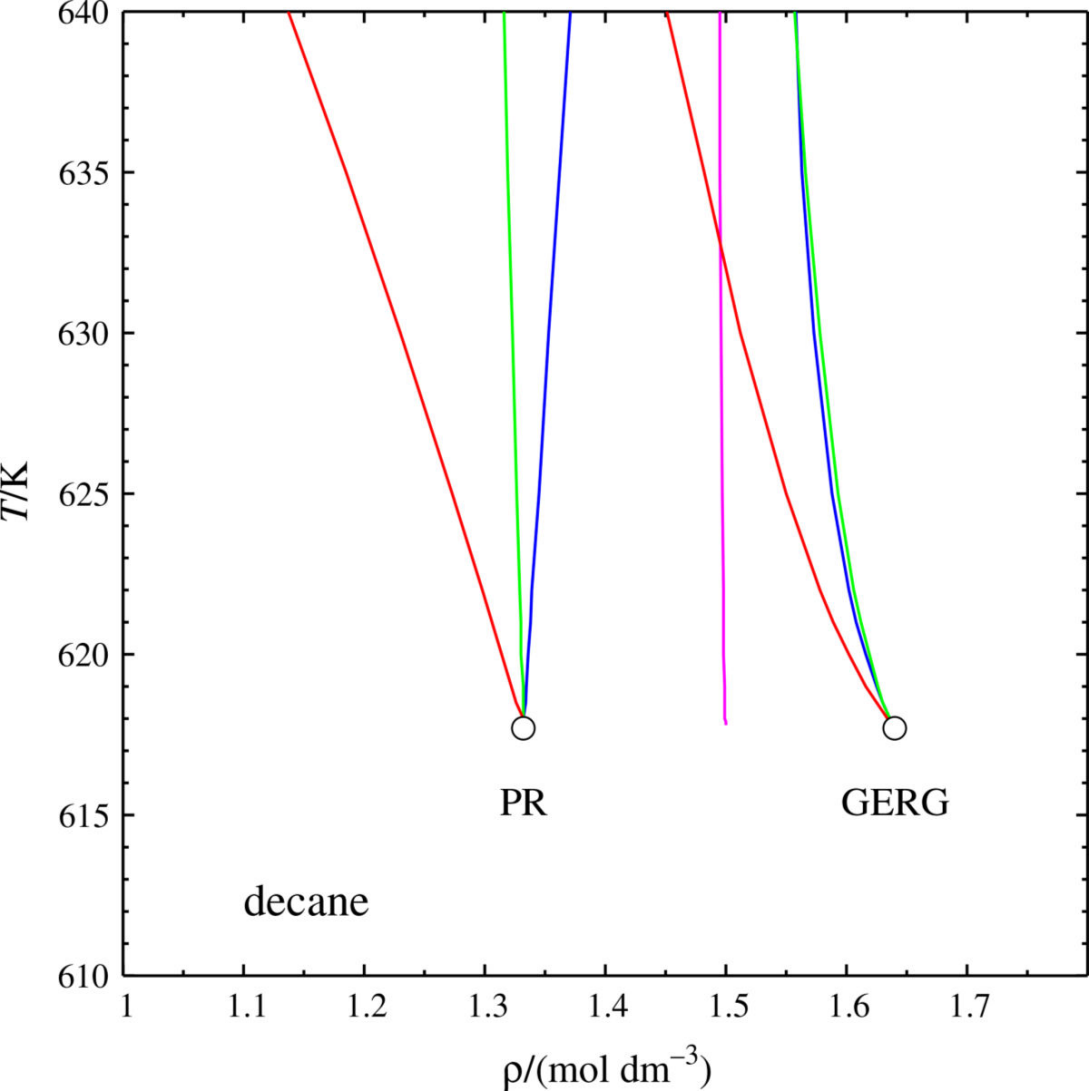
Widom curves of decane on the temperature–density plane, calculated with the Peng–Robinson and the GERG equations of state. Red: isothermal compressibility *κ*_*T*_, green: isobaric expansivity *α*_*p*_, blue: isobaric heat capacity *C*_*p*_, magenta: isochoric heat capacity *C*_*V*_

Figure 9 shows some Widom curves for decane, calculated with the Peng–Robinson and the GERG-2008 equations of state. The PR equation yields a too low critical density, therefore its curves appear shifted to the left. The Widom curves of the isobaric expansivity, *α*_*p*_, and the isobaric heat capacity, *C*_*p*_, remain close to each other, as expected, whereas the curve of the isothermal compressibility, *κ*_*T*_, veers off towards lower densities with a concave curvature. There is no Widom curve for the isochoric heat capacity, *C*_*V*_, because cubic equations of state must have classical critical exponents (in this case *α* = 0).

In contrast to this, the GERG equation does have a *C*_*V*_ Widom curve—but it does not originate at the critical point. This is clearly an artifact. The *κ*_*T*_ Widom curve is left of the other two, as expected—but its curvature is convex, not concave. In other words: the GERG equation exhibits unphysical behavior in a very sensitive region.

## 5 Conclusion

When critical curves of binary fluid mixtures are calculated with the present GERG models—GERG-2004 and GERG-2008—there is often a distortion in the vicinity of the critical point of the less volatile component. Particularly, if the critical temperatures of the mixture components differ much, this distortion can become so large that the critical curve develops a temperature maximum. Isothermal *px* diagrams calculated in the vicinity of the maximum exhibit impossible shapes of the phase envelopes. In other words: the GERG model can predict physically unreasonable phase equilibria.

Test calculations with an extended van der Waals equation of state capable of approximating the critical isotherm of a real fluid showed that wrong phase diagram classes, or even unphysical shapes of the mixture critical curves resulted as soon as enough correction terms were activated to achieve a quantitative agreement.

We therefore conjecture that a GERG equation-like method for predicting thermodynamic properties of mixtures should be built with simpler pure-fluid equations of state exhibiting classical critical exponents, and that corrections for critical anomalies—which are different for pure fluids and for mixtures—should be introduced at a later stage.

## Figures and Tables

**Table 1:** Parameters of the equation of state Eq. (11)

*k*_max_	0	4	6	8

Methane				
T* · K^−1^	204.816535	204.865410	204.966907	204.965915
v* · cm^−3^ · mol^−1^	17.9985080	17.9957587	17.9901196	17.9901751
*c*	3.69778623	3.69934884	3.70268257	
*w*		1.95327250	0.144489799	0.742822019
*p*_4_		−0.00389354071	0.00308448370	0.00479901118
*p*_5_			−0.00433154314	−0.00473229504
*p*_6_			−0.00827940452	−0.0158992309
*p*_7_				−0.00259676851
*p*_8_				0.0132045635
Hydrogen				
T* · K^−1^	35.6238306	35.6321636	35.6501026	35.6499400
v* · cm^−3^ · mol^−1^	11.1080159	11.1057029	11.1007273	11.1007724
*c*, *w*, *p*_4...8_	Same as for methane			

## Data Availability

Not applicable.Code availability *ThermoC* thermodynamic program package, available from the corresponding author upon request.

## Footnotes

1 Recent studies indicate that the divergence of *C*_*V*_ is logarithmic, i.e., *α* = 0 while *C*_*V*_ is no longer an intensive property. But the distinction is not relevant for this work.

## Symbols

*A*: Helmholtz energy
*B_X_*: Critical amplitude for property *X*
*C_p_, C_V_*: Isobaric, isochoric heat capacity
*c, w, p_k_*: Parameters of critical-anomaly correction
*k_ij_*: Adjustable parameter in mixing rule Eq. (6)
*N*: Number of components
*p*: Pressure
*R*: Gas constant
*S*: Entropy
*T*: Temperature
*T**: Characteristic temperature
***u***_min_: Eigenvector associated with *λ*_min_
*v**: Characteristic volume
***x***: Vector of mole fractions, ***x*** = (*x*_1_,…, *x_N_*)
*α,…, δ*: Critical exponents
*α*^r^: Dimensionless residual Helmholtz energy ($\alpha_{0i}^{\text{r}}$: of component i,$\alpha_{ij}^{\text{r}}$: of component pair *ij* within the departure function)
*α_p_*: Isobaric thermal expansivity
*β_X,ij_, γ_X,ij_*: GERG parameters for the reducing property *X*_r_ (*X* = *T*, *V*)
*κ_T_*: Isothermal compressibility
*λ*_min_: Lowermost eigenvalue of **Ψ**
*ρ*: Molar density
***ρ***: Vector of molar concentrations, ***ρ*** = *ρ****x***
*τ*: Reciprocal reduced temperature
Ψ: Helmholtz energy density, **Ψ** = *ρ**A*_m_
**Ψ**: Hessian matrix of **Ψ**(***ρ***)
ω: Reduced density
